# Supplementary material for: Dose-dependent interferon programs in myeloid cells after mRNA and adenovirus COVID-19 vaccination
Source: bioRxiv. 2025 Aug 18:2025.08.15.668720. Preprint. [Version 1] doi: 10.1101/2025.08.15.668720 (PMC12393322; doi:10.1101/2025.08.15.668720)
Supplement: Supplement 8 [file NIHPP2025.08.15.668720v1-supplement-8.pdf]

bioRxiv preprint doi: https://doi.org/10.1101/2025.08.15.668720; this version posted August 18, 2025. The copyright holder for this preprint (which was not certified by peer review) is the author/funder, who has granted bioRxiv a license to display the preprint in perpetuity. It is made available under aCC-BY 4.0 International license.

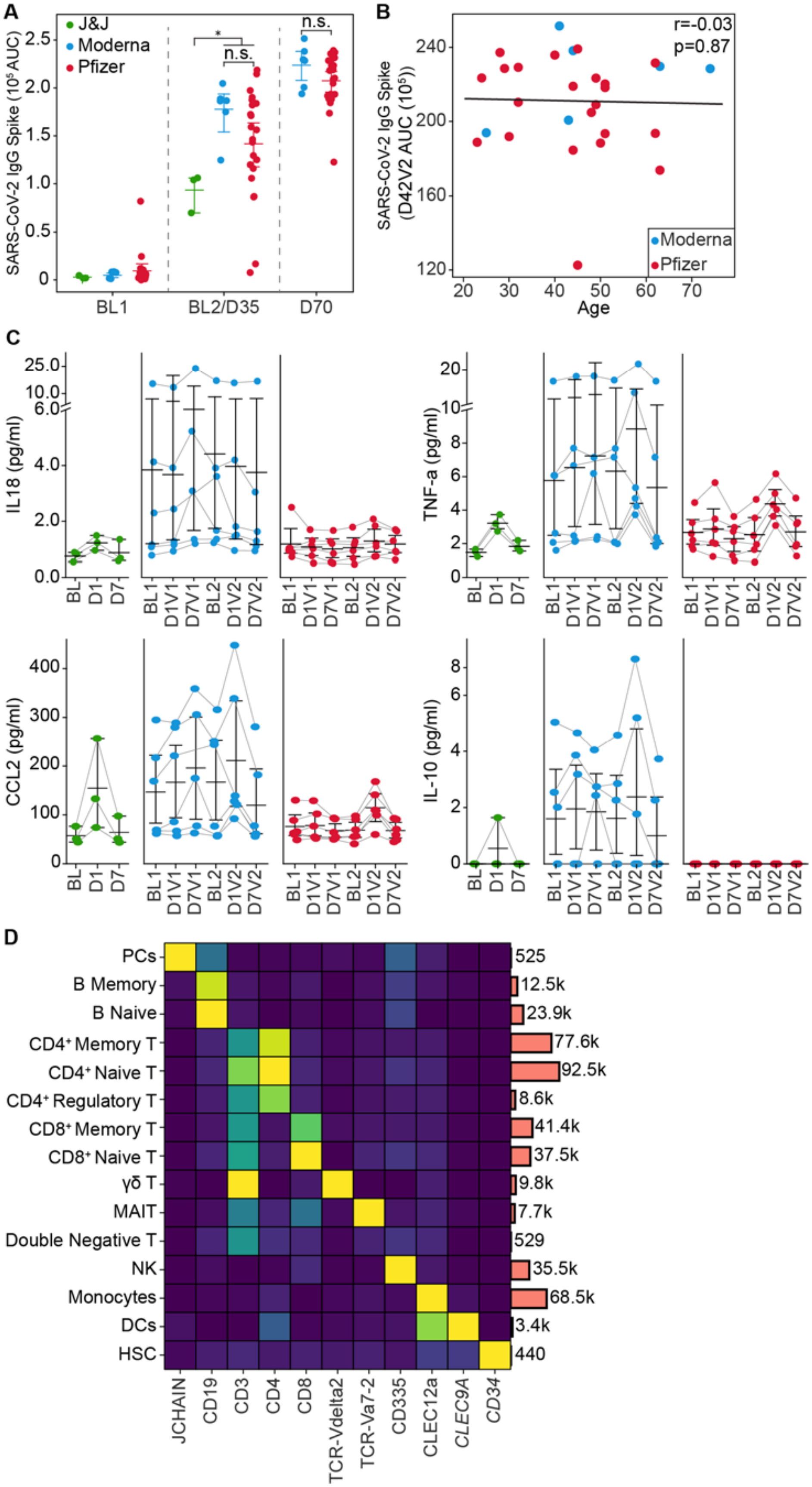

bioRxiv preprint doi: https://doi.org/10.1101/2025.08.15.668720; this version posted August 18, 2025. The copyright holder for this preprint (which was not certified by peer review) is the author/funder, who has granted bioRxiv a license to display the preprint in perpetuity. It is made available under aCC-BY 4.0 International license.

Figure S1: Antibody titer and cytokine level after adenovirus and mRNA vaccination
(A) IgG titers against SARS-CoV-2 spike protein measured by ELISA. mRNA vaccines elicit higher antibody titer compared to J&J. (**B)** The correlation plot with age and antibody titer for mRNA vaccines. Pearson's correlation was used to calculate statistics. **C)** IL-8, TNFα, CCL2, and IL-10 cytokine levels, quantified by ELLA. **D)** The heatmap shows the marker genes for single-cell annotations. (A) Statistical comparisons were performed using the Mann-Whitney test: n.s.: non-significant, *$P < 0.05$, **$P < 0.01$, ***$P < 0.001$, ****$P < 0.0001$.

bioRxiv preprint doi: https://doi.org/10.1101/2025.08.15.668720; this version posted August 18, 2025. The copyright holder for this preprint (which was not certified by peer review) is the author/funder, who has granted bioRxiv a license to display the preprint in perpetuity. It is made available under aCC-BY 4.0 International license.

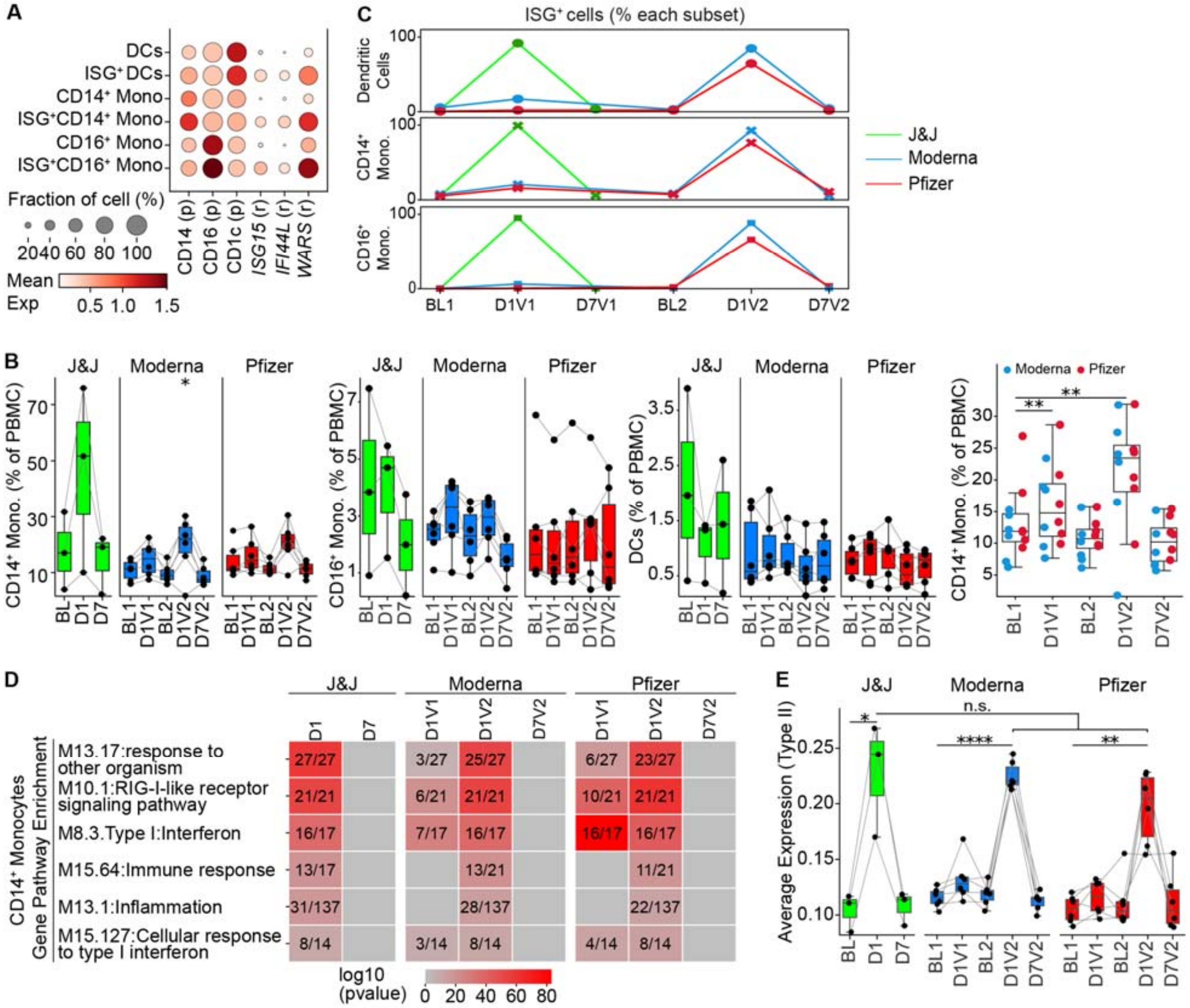

Figure S2: Adenovirus and mRNA vaccines response in CD14⁺ monocytes.

A) The average expression of marker genes in each myeloid and ISG subsets. **B)** The percentage of CD14⁺ monocytes, CD16⁺ monocytes and dendritic cells (DCs) in total PBMC (left). Significant expansion of the CD14⁺ monocytes upon mRNA vaccination (right). (C) The percentage of ISG subsets within each lineage for each vaccine. **D)** The heatmap shows the top 6 enriched pathways obtained by over-representation analysis using the Blood3GenModule. The number inside indicates the number of overlaps for the respective module. **E)** Type-II interferon expression score calculated from manually curated list (n=51). (B) Statistical comparisons were performed using the one-sided Wilcoxon test, (E) Statistical significance between timepoints were performed using two-tailed paired t-test, statistical significance between vaccine was performed using Mann-Whitney test: n.s. non-significant, *$P < 0.05$, **$P < 0.01$, ***$P < 0.001$, ****$P < 0.0001$.

bioRxiv preprint doi: https://doi.org/10.1101/2025.08.15.668720; this version posted August 18, 2025. The copyright holder for this preprint (which was not certified by peer review) is the author/funder, who has granted bioRxiv a license to display the preprint in perpetuity. It is made available under aCC-BY 4.0 International license.

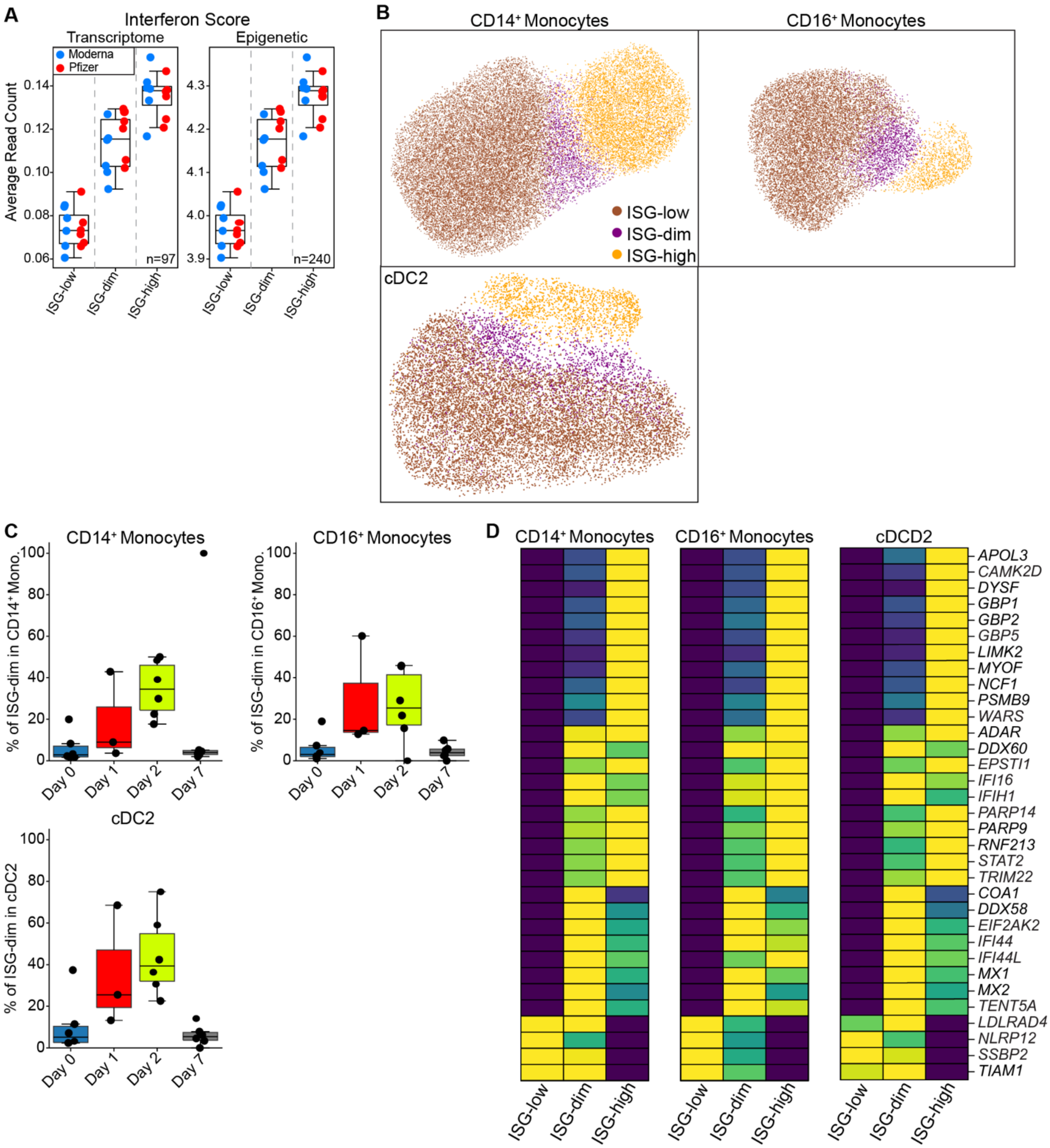

Figure S3: Distinct ISG subset signature in publicly available data from donors vaccinated with BNT162b2.

(A) Interferon scores for cells in each ISG state. **(B)** UMAP representation of ISG-low, ISG-dim, and ISG-high subsets in CD14^{+} monocytes, CD16^{+} monocytes and cDC2 cell types. **(C)** ISG-dim cell percentage within respective cell populations across different timepoints. **(D)** The heatmap displays the expression levels of marker genes for ISG states in CD14^{+} monocytes, CD16^{+} monocytes, and cDC2s.

bioRxiv preprint doi: https://doi.org/10.1101/2025.08.15.668720; this version posted August 18, 2025. The copyright holder for this preprint (which was not certified by peer review) is the author/funder, who has granted bioRxiv a license to display the preprint in perpetuity. It is made available under aCC-BY 4.0 International license.

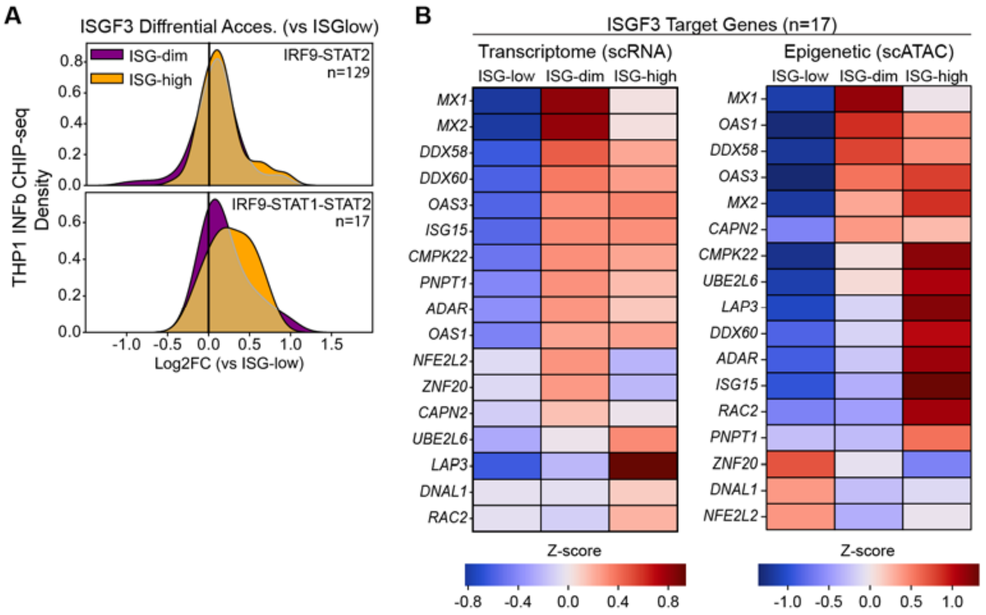

Figure S4: ISGF3 Transcription factor epigenetic dynamic changes

A) Kernel density estimation plots show the distribution of accessibility of transcription factor binding sites in ISG-dim and ISG-high compared to the ISG-low subset. IRF9-STAT2 and IRF9-STAT1-STAT2 (ISGF3) peaks were identified by intersecting each TF motif of overlapping peaks of CHIP-seq data. **B)** The heatmap shows the expression and accessibility of the genomic region targeted by the ISGF3 complex in three ISG states.

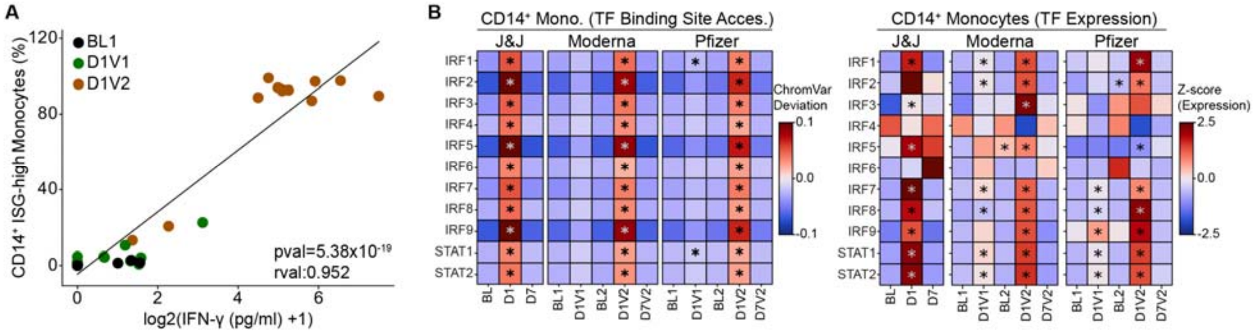

Figure S5: IFNG cytokine levels correlates with ISG-high CD14$^+$ Monocyte

A) Pearson correlation between ISG-high state percentage in CD14+ monocytes and IFN-γ cytokine levels per sample. **B)** Longitudinal binding site activity of the IRFs and STATs calculated by ChromVAR (left); corresponding gene expression levels (right) of same TFs (right). (B) Statistical significance between timepoints were performed using one-tailed paired t-test, $*P < 0.05$.
